# RACK1 links immune signaling and translation during host-pathogen interactions

**DOI:** 10.1080/21505594.2026.2757928

**Published:** 2026-11-02

**Authors:** Rongrong Ding, Yunnuo Shi, Noah R. Martin, Siyu Huo, Xinwei Jiang, Gregory D. Fairn, Zhenyu Cheng, Yiming Yang

**Affiliations:** a Department of Cell Biology and Medical Genetics, College of Basic Medical Sciences, Jilin University, Changchun, Jilin, China; b Department of Microbiology and Immunology, Dalhousie University, Halifax, NS, Canada; c Department of Pathology, Dalhousie University, Halifax, NS, Canada; d Department of Biochemistry and Molecular Biology, Dalhousie University, Halifax, NS, Canada

**Keywords:** RACK1, host-pathogen interaction, immune signaling, translational regulation, innate immunity

## Abstract

The outcome of an infectious disease results from a dynamic interplay between host immune responses and pathogen virulence mechanisms. RACK1 is a highly conserved scaffold protein that integrates signal transduction, translational control, and cellular stress responses. Emerging evidence positions RACK1 as a central hub at the host–pathogen interface. It coordinates host immune responses, yet pathogens also exploit it to promote their survival and persistence. However, current knowledge of RACK1 in host–pathogen interactions remains fragmented, with most studies focusing on specific pathogens. This review summarizes the structural and cellular functions of RACK1 in host defense, examines the molecular strategies employed by pathogens to exploit RACK1-dependent pathways during infection, and integrates findings across diverse infectious systems to provide a comprehensive overview of RACK1-mediated host–pathogen interactions. We further discuss the role of RACK1 in infection outcomes, its therapeutic potential, and key directions for future research.

## Introduction

Host–pathogen interactions represent a dynamic interplay between host defense mechanisms and pathogen virulence strategies and are central to the initiation, progression, and resolution of infectious diseases. Receptor for activated C kinase 1 (RACK1) is a highly conserved WD40-repeat scaffold protein that interacts with numerous signaling molecules and participates in pathways regulating inflammation, cellular stress, survival, and cytoskeletal organization. In addition to these signaling functions, RACK1 is an integral component of the 40S ribosomal subunit and contributes to the regulation of protein translation [1].

The functional versatility of RACK1 has attracted increasing attention in the context of host–pathogen interactions. Depending on the biological context, RACK1 can participate in inflammatory and innate immune signaling, cellular stress responses, cytoskeletal remodeling, pathogen entry and replication, and protein synthesis. Conversely, microbial proteins can interact with RACK1 or manipulate RACK1-containing complexes to alter host signaling and exploit cellular processes required for infection. RACK1 therefore cannot be simply classified as either host-protective or infection-promoting; its function depends on the signaling pathway involved, the cellular context, and the infection strategy employed by the pathogen. Recent reviews have comprehensively summarized the roles of RACK1 in bacterial and viral infections [2,3]. However, increasing recognition of RACK1 as both a signaling scaffold and a ribosome-associated regulatory protein raises a broader mechanistic question: how are these two functional identities integrated during host–pathogen interactions?

In 2012, Gibson highlighted two largely disconnected areas of RACK1 research: one centered on signaling and cellular regulation and the other on translation and ribosome biology [4]. In this review, the author proposed that RACK1 may provide an important molecular link between these processes. Accumulating evidence supports this concept, indicating that ribosome association does not simply transform RACK1 from a signaling scaffold into a passive structural component of the translational machinery. Instead, ribosome-associated RACK1 retains its scaffolding capacity and can provide signaling proteins with direct access to the translational apparatus. A well-characterized example involves RACK1-associated protein kinase C (PKC), which promotes eIF6 phosphorylation and thereby facilitates the formation of translationally active 80S ribosomes. Similarly, RACK1-associated Src and JNK connect signaling pathways with selective mRNA translation and protein quality control, respectively.

This signaling–translation interface may be particularly important during infection, when pathogens simultaneously reprogram host signaling pathways and protein synthesis [5]. In this review, we examine RACK1-mediated host–pathogen interactions by integrating these historically separated aspects of RACK1 biology. We first discuss the structural and cellular features that enable RACK1 to function both within and outside the ribosome, and then consider how host and pathogen-derived signals engage these distinct functional states. Particular emphasis is placed on how RACK1-dependent signaling may intersect with translational regulation during infection. By bridging signaling, ribosome biology, and infection research, this review aims to provide a unified mechanistic framework for understanding the context-dependent roles of RACK1 in host–pathogen interactions and to highlight key unanswered questions regarding how the RACK1 signaling–translation interface influences infection outcomes.

## Structural and cellular basis of RACK1

### Seven-WD40 β-propeller structure

RACK1, formerly known as GNB2L1 (guanine nucleotide-binding protein subunit beta-2-like 1), belongs to the tryptophan–aspartic acid repeat (WD40) protein family [6,7]. The designation GNB2L1 originated from its sequence similarity to G-protein β subunits, whereas the name RACK1 became widely adopted following its identification as a receptor for activated protein kinase C. RACK1 was first cloned from a chicken liver cDNA library in 1989 [8] and was subsequently shown to be highly conserved across eukaryotic species [9]. From unicellular eukaryotes to plants and mammals, RACK1 orthologues exhibit substantial sequence conservation, with reported sequence identities ranging from 43% to 76% [10,11]. This evolutionary conservation is further illustrated by multiple sequence alignment of RACK1 orthologues from representative eukaryotic species (Figure 1).

**Figure 1.**
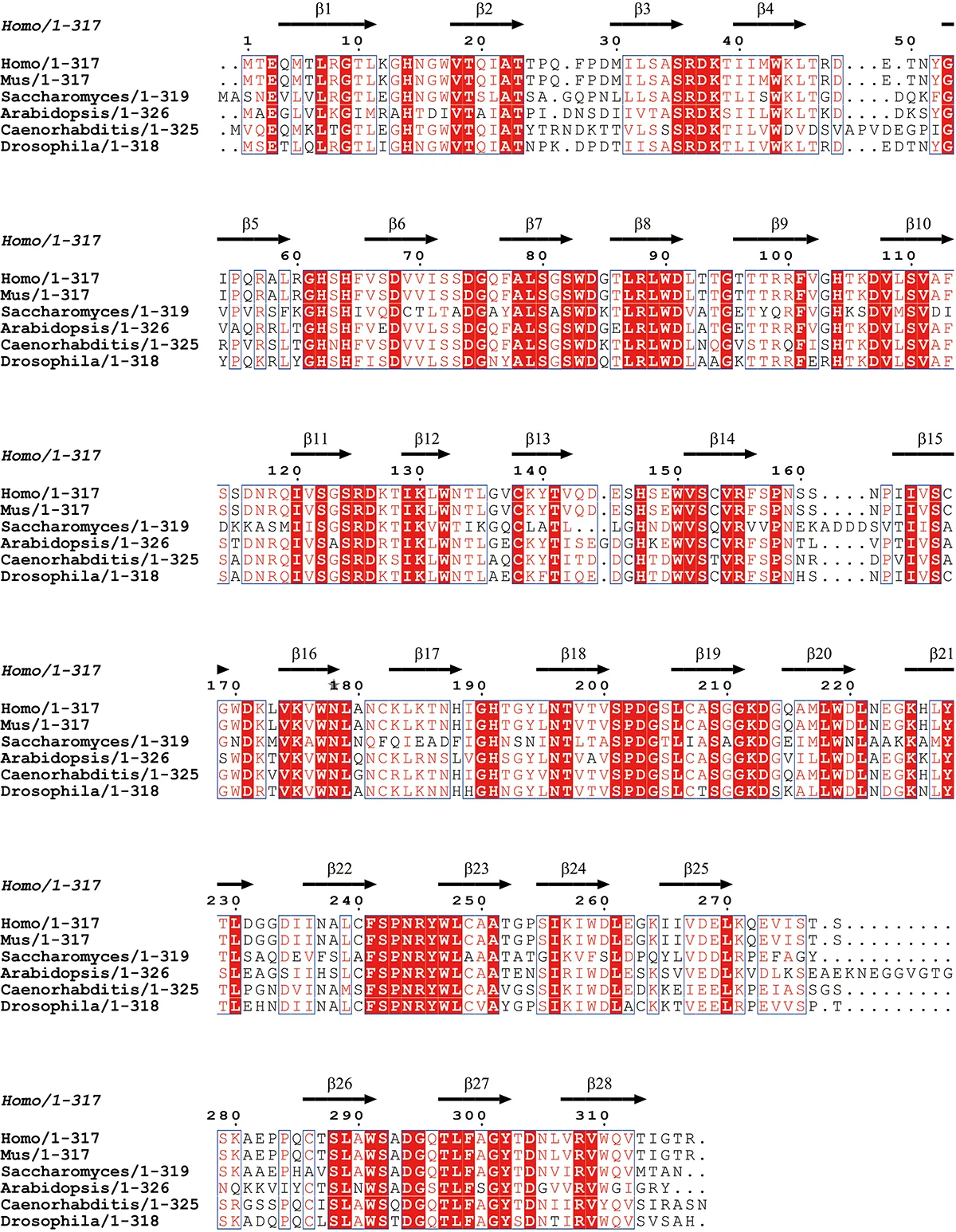
Multiple sequence alignment of RACK1 orthologues across representative eukaryotic species. RACK1 protein sequences from *Homo sapiens*, *Mus musculus*, *Saccharomyces cerevisiae*, *Arabidopsis thaliana*, *Caenorhabditis elegans*, and *Drosophila melanogaster* were aligned. WD40 repeats and secondary-structure elements are indicated above the alignment. Highly conserved residues are highlighted in red, illustrating the substantial sequence conservation of RACK1 across eukaryotic species.

WD40 repeats typically comprise approximately 40–60 amino acids and contain a conserved WD motif. Each repeat contributes to a blade composed of four antiparallel β-strands, and multiple repeats assemble into a stable β-propeller architecture [12,13]. RACK1 contains seven WD40 repeats that fold into a characteristic ring-shaped, seven-bladed β-propeller. This architecture generates multiple protein-binding surfaces on the upper and lower faces, lateral regions, and around the central pore, providing a structural basis for the ability of RACK1 to interact with diverse binding partners [13].

The crystal structure of human RACK1, resolved at 2.45 Å, confirmed its characteristic seven-bladed β-propeller architecture and provided important structural insight into its scaffolding properties [14]. Conserved tryptophan residues are largely buried between adjacent blades, where they contribute to inter-blade packing and stabilization of the structural core, whereas more variable residues are exposed on the protein surface and may contribute to partner-specific interactions [15]. Moreover, folding of the β-propeller brings residues that are distant in the primary sequence into spatial proximity, thereby generating composite binding surfaces. Peptide-based studies have identified several regions involved in PKC binding, including residues 99–108 within β-strand D of blade II and the connecting loop between blades II and III, as well as residues 234–241 encompassing part of blade VI and its preceding loop [16]. WD repeats 5–7 constitute a major interaction region for PDE4D5, with multiple mutations that disrupt PDE4D5 binding clustering on a discrete surface of the RACK1 β-propeller [17]. Together, these structural features enable RACK1 to engage diverse binding partners and assemble distinct signaling complexes. The structural organization of the RACK1 β-propeller is illustrated in Figure 2.

**Figure 2.**
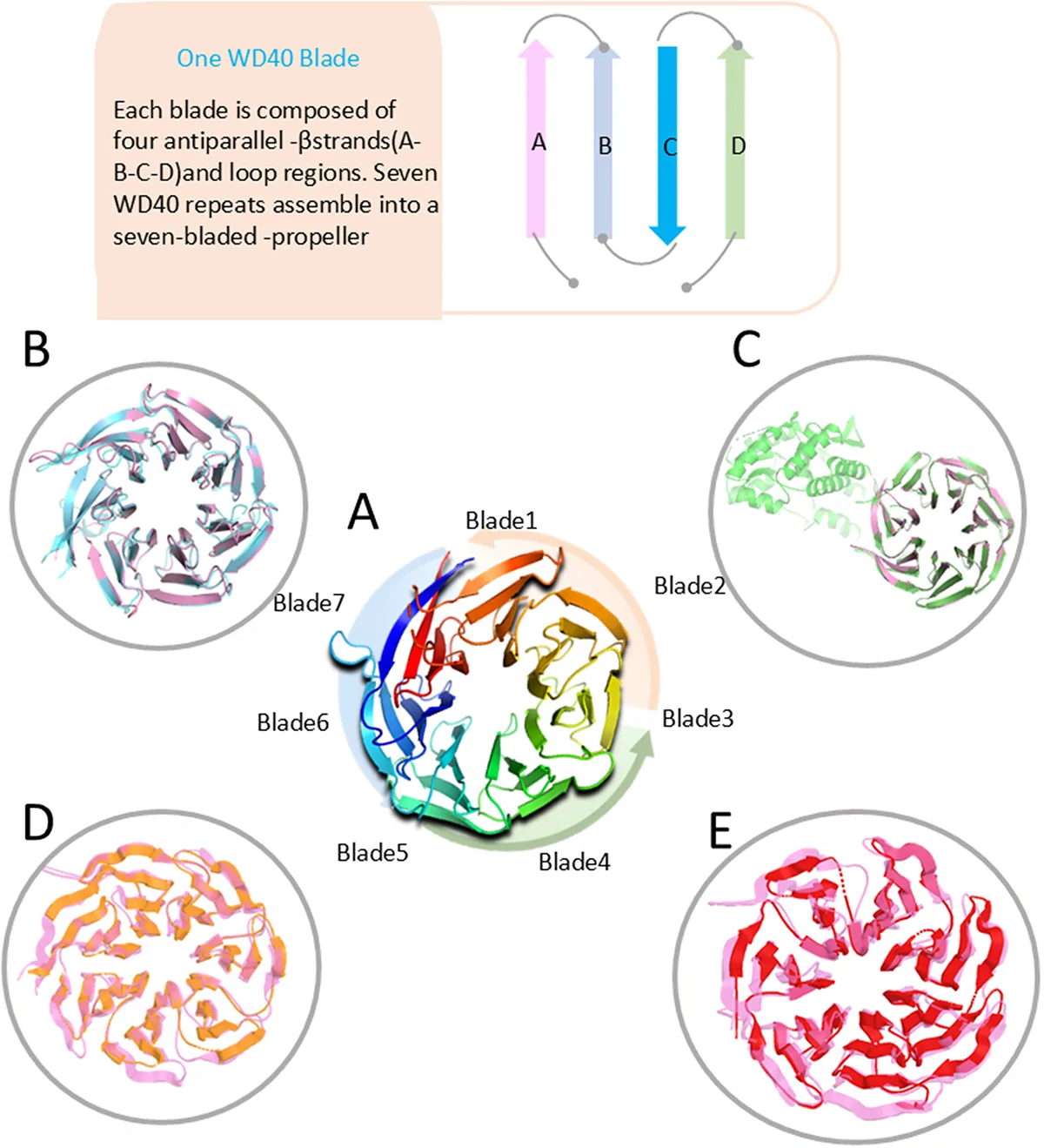
Structural organization and conserved architecture of RACK1. (A) Crystal structure of human RACK1 (PDB: 4AOW), showing the characteristic seven-bladed β-propeller formed by seven WD40 repeats. The inset illustrates a schematic representation of a single WD40 blade, composed of four antiparallel β-strands connected by loop regions.(B) Structural superposition of human RACK1 and *Saccharomyces cerevisiae* RACK1/Asc1 (PDB: 3FRX).(C) Structural superposition of human RACK1 and *Arabidopsis thaliana* RACK1A (PDB: 3DM0), demonstrating conservation of the WD40 β-propeller architecture.(D) Structural superposition of human RACK1 (pink; PDB: 4AOW) and the *Tetrahymena thermophila* RACK1 homolog (orange; PDB: 2XZN), further illustrating the conserved seven-bladed β-propeller architecture.(E) Structural superposition of human RACK1 (pink; PDB: 4AOW) and Gβ1 (red; PDB: 1TBG), highlighting the conserved β-propeller fold shared by WD40-repeat proteins.

### RACK1 distribution and subcellular localization

RACK1 is broadly expressed across mammalian tissues, although its abundance varies among organs and cell types [18]. In the mouse nervous system, RACK1 expression is developmentally regulated and exhibits regional enrichment in the adult brain, including the cerebral cortex, hippocampus, and cerebellum. Consistent with its widespread expression, the Human Protein Atlas classifies RACK1 as a gene with low tissue specificity and reports both nucleoplasmic and cytosolic localization in human cell lines [19].

Consistent with its function as a non-enzymatic scaffold, RACK1 can be recruited to distinct protein complexes and subcellular compartments in a context-dependent manner. During integrin-dependent cell adhesion, RACK1 associates with adhesion-related complexes and accumulates in nascent peripheral focal complexes at sites of membrane protrusion [20]. Thus, rather than remaining uniformly distributed in the cytoplasm, RACK1 can be dynamically recruited to spatially restricted signaling platforms. In endothelial cells, RACK1 also participates in an eIF6–RACK1–ERK1/2–FAK mechanocomplex that supports localized ERK1/2 activation and force-induced focal-adhesion remodeling [21].

RACK1 localization is also dynamically regulated in response to cellular stress. Under conditions such as hypoxia, heat shock, and arsenite exposure, RACK1 can be recruited to cytoplasmic stress granules, thereby reducing its association with MTK1 and attenuating stress-responsive MAPK signaling [22]. RACK1 has also been identified among O-GlcNAc-modified proteins associated with arsenite-induced stress granules [23]. More recently, PARP14-mediated mono-ADP-ribosylation (MARylation) of RACK1 was shown to promote its colocalization with G3BP1, eIF3η, and 40S ribosomal proteins in stress granules in ovarian cancer cells [1], illustrating how post-translational modification can regulate the spatial organization of RACK1. Beyond cytoplasmic compartments, activation of the cAMP/PKA pathway promotes RACK1 translocation to the nucleus, where its interaction with 14–3-3ζ is required for cAMP-induced BDNF transcription [24]. Collectively, these findings indicate that RACK1 exists in multiple spatially and functionally distinct pools and that its subcellular distribution can be dynamically remodeled by extracellular signals, mechanical cues, and cellular stress.

## RACK1 in organizing host signaling during infection

### RACK1 as a signaling scaffold

RACK1 regulates the subcellular localization, activity, stability, and interactions of numerous binding partners [7]. By simultaneously engaging multiple proteins, RACK1 functions as a molecular scaffold that facilitates the spatial and temporal organization of signaling complexes and modulates their downstream outputs [25]. A classical example is its interaction with activated PKCβII. RACK1 recruits PKCβII to specific subcellular sites and brings the kinase into proximity with its substrates, thereby facilitating PKC-dependent phosphorylation and downstream signal transduction [6,26].

Beyond PKC signaling, RACK1 participates in the regulation of multiple signaling pathways, including mitogen-activated protein kinase/c-Jun N-terminal kinase (MAPK/JNK), cyclic AMP-specific phosphodiesterase 4D5 (PDE4D5), Src, protein phosphatase 2A (PP2A), focal adhesion kinase (FAK), insulin-like growth factor 1 receptor (IGF-1 R), N-methyl-D-aspartate (NMDA) receptor, Wnt/β-catenin signaling, common β-chain cytokine receptor signaling, and type I interferon receptor signaling [22,27–38]. Through these interactions, RACK1 coordinates signaling networks involved in cellular stress responses, inflammatory signaling, and immune regulation. This scaffolding capacity provides an important mechanistic basis for the context-dependent roles of RACK1 during host–pathogen interactions, in which both host-derived and pathogen-derived signals can converge on RACK1-associated signaling complexes.

### RACK1 in host innate immune signaling

During infection, RACK1 participates in receptor-proximal inflammatory signaling downstream of pattern-recognition receptors (PRRs). Following recognition of pathogen-associated molecular patterns (PAMPs), adaptor proteins and kinases such as MyD88, TRAF6, and TAK1 are rapidly recruited to receptor-associated signaling complexes, leading to activation of the IKK complex and MAPK cascades [37,39–42]. Through the multiple interaction surfaces provided by its WD40 β-propeller, RACK1 can facilitate the spatial organization of signaling components associated with the NF-κB and MAPK pathways [22,41].

RACK1 is particularly linked to the JNK/SAPK branch of MAPK signaling. RACK1 deficiency has been reported to attenuate JNK/SAPK activation, suggesting that RACK1 contributes to the assembly and functional coordination of stress-responsive MAPK signaling complexes. Mechanistically, RACK1 facilitates PKC-mediated phosphorylation of JNK at Ser129. Although phosphorylation at this site is insufficient to activate JNK directly, it enhances subsequent phosphorylation by MKK4 and/or MKK7, thereby amplifying stress-induced JNK signaling [6,29].

Importantly, the effects of RACK1 on infection-associated NF-κB and MAPK signaling are highly context dependent. During classical swine fever virus (CSFV) infection, RACK1 expression is reduced in infected PK-15 cells, and RACK1 knockdown is associated with increased viral infection and enhanced NF-κB activation, whereas RACK1 overexpression produces the opposite effects [43,44]. In contrast, RACK1 appears to facilitate porcine reproductive and respiratory syndrome virus (PRRSV) infection in several experimental models. RACK1 has been reported to support efficient PRRSV replication, participate in a positive-feedback loop involving NF-κB and integrin β3, and promote viral infection through ERK1/2 activation [44–46]. These contrasting observations illustrate that the influence of RACK1 on inflammatory signaling depends strongly on the pathogen and cellular context. Because much of this evidence derives from established cell-line models, however, whether similar regulatory patterns operate in primary cells and *in vivo* remains to be established.

RACK1 also regulates antiviral signaling downstream of cytosolic RNA sensors. In the RIG-I-like receptor (RLR)–MAVS pathway, efficient signal amplification depends on the recruitment of downstream effectors, including TRAF proteins, to the MAVS signaling platform. RACK1 has been shown to modulate MAVS-associated signaling complexes by influencing MAVS–TRAF interactions, thereby altering MAVS-dependent antiviral signal transduction [47]. In addition, activation of AMP-activated protein kinase (AMPK) during viral infection promotes phosphorylation of RACK1 at Thr50, strengthens its interaction with IRF3 and IRF7, and restricts IRF3/IRF7 phosphorylation. This suppresses transcriptional activation of type I interferon genes and reduces IFN-α/IFN-β production [48]. Thus, RACK1 can regulate antiviral signaling at multiple levels, from MAVS-associated complex assembly to downstream activation of interferon-regulatory factors.

Following the induction of type I interferons, amplification of the antiviral response depends on signaling through the type I interferon receptor and the JAK1/TYK2–STAT pathway [49–52]. RACK1 constitutively associates with the type I interferon receptor subunit IFNAR2 and, following receptor activation, participates in a signaling complex containing IFNAR2, JAK1, TYK2, and STAT1, thereby facilitating TYK2-dependent phosphorylation of STAT1 [37,53]. Disruption of RACK1 binding to the Box2 region of IFNAR2 markedly reduces tyrosine phosphorylation of STAT1 and STAT2 and diminishes protection against the cytopathic effects of encephalomyocarditis virus [53]. These findings support a role for RACK1 as a scaffold that stabilizes receptor-associated signaling complexes and promotes efficient transmission of type I interferon signals.

RACK1 also participates in DNA-sensing antiviral pathways. In the cGAS–STING pathway, RACK1 associates with STING and enhances STING-dependent type I interferon signaling, further supporting the concept that RACK1 contributes to the organization of innate antiviral signaling complexes. Collectively, these studies indicate that RACK1 acts at multiple levels of innate immune signaling, while the direction and magnitude of its effects are determined by the specific receptor pathway, cellular environment, and pathogen involved.

### RACK1 in inflammasome activation and inflammatory responses

Inflammasomes are cytosolic signaling platforms that promote caspase-1 activation, maturation and secretion of IL-1β and IL-18, and gasdermin D (GSDMD)-mediated pyroptosis [54,55]. Activation of the NLRP3 inflammasome is commonly described by a two-signal model. Signal 1, or priming, activates NF-κB and induces the expression of NLRP3 and pro-IL-1β, whereas Signal 2 is provided by cellular stress signals such as ionic imbalance, mitochondrial dysfunction, increased reactive oxygen species (ROS), and lysosomal damage [42,56–58]. These stimuli promote NLRP3 activation and oligomerization, followed by recruitment of the adaptor ASC and pro-caspase-1 to assemble the functional inflammasome complex.

RACK1 participates in NLRP3 inflammasome regulation at both the priming and assembly stages. Consistent with its role in NF-κB signaling described above, RACK1 can contribute to inflammasome priming by promoting the expression of NLRP3 and pro-IL-1β in relevant infection settings. RACK1 can also physically associate with NLRP3 and function as a scaffold that facilitates inflammasome assembly [59]. Mechanistically, RACK1 has been proposed to promote the transition of NLRP3 from an autoinhibited to an activation-competent conformation and to support NLRP3 oligomerization, ASC recruitment, and caspase-1 activation, ultimately enhancing IL-1β release and GSDMD-mediated pyroptosis [60].

This regulatory role has been demonstrated during *Pasteurella multocida* infection. RACK1 promotes inflammasome activation through both NF-κB-dependent priming and assembly of the NLRP3–NEK7 complex. RACK1 knockdown reduces *P. multocida*-induced NF-κB activation, decreases the expression of NLRP3, pro-IL-1β, and caspase-1, and suppresses ASC speck formation, whereas RACK1 overexpression enhances these responses. RACK1 also colocalizes and interacts with NLRP3 and NEK7. Notably, inhibition of K^+^ efflux weakens formation of the RACK1–NLRP3–NEK7 complex, suggesting that RACK1-dependent inflammasome assembly is linked to K^+^ efflux [59].

RACK1-dependent inflammasome regulation is also evident during *Mycobacterium tuberculosis* infection. The *M. tuberculosis*-secreted protein EST12 binds to RACK1 in macrophages, and the EST12–RACK1 complex recruits the deubiquitinating enzyme UCHL5. UCHL5 promotes removal of K48-linked ubiquitin chains from NLRP3, thereby limiting its proteasomal degradation and increasing NLRP3 abundance. This, in turn, enhances activation of the NLRP3–caspase-1/11–GSDMD–IL-1β pathway [61]. These findings suggest that RACK1 can serve as a molecular bridge through which pathogen-derived effector proteins engage and modulate the host inflammasome machinery.

Collectively, these studies indicate that RACK1 can coordinate both the priming and assembly phases of NLRP3 inflammasome activation during infection. Its contribution may therefore extend beyond general inflammatory signaling to the spatial organization and stabilization of inflammasome-associated protein complexes. However, whether these mechanisms are broadly conserved across different pathogens, immune-cell populations, and *in vivo* infection settings remains to be determined.

### RACK1 in cellular stress responses

Beyond its role in innate immune signaling, RACK1 participates in cellular stress responses by organizing stress-responsive protein complexes and regulatory pathways. RACK1 has been implicated in the regulation of autophagy through its interaction with the autophagy protein ATG5. This interaction is enhanced under autophagy-inducing conditions, including nutrient starvation and mTOR inhibition. Depletion of RACK1 or disruption of the RACK1–ATG5 interaction attenuates autophagic activation, supporting a scaffolding role for RACK1 in coordinating autophagic stress responses [62].

RACK1 also contributes to the regulation of oxidative stress in a context-dependent manner. In hepatocellular carcinoma cells, RACK1 interacts with and stabilizes carbonyl reductase 1 (CBR1), thereby limiting ROS accumulation and cell death induced by TNF-α or H_2_O_2_ [63]. In neurons, RACK1 forms a complex with DJ-1, and depletion of RACK1 compromises DJ-1-mediated protection against H_2_O_2_-induced apoptosis [64]. These findings suggest that RACK1 can enhance cellular resistance to oxidative damage by stabilizing stress-protective protein complexes, although its effects may vary according to cell type and stress conditions. Because these mechanisms have been characterized predominantly in noninfectious models, their relevance to pathogen-induced cellular stress remains to be established.

### RACK1 in immune-cell homeostasis and adaptive immunity

Beyond its immediate effects on inflammatory signaling, RACK1 also contributes to the development, maintenance, and function of immune-cell populations involved in host defense. In the myeloid compartment, RACK1 deficiency results in a reduction in mature alveolar macrophages and increases susceptibility to *Streptococcus pneumoniae* infection in mice. Mechanistically, RACK1 binds to and stabilizes PPARγ, limiting its ubiquitination and degradation and thereby supporting alveolar macrophage development and antimicrobial function [65]. These findings extend the role of RACK1 beyond acute signaling events to the maintenance of innate immune-cell homeostasis.

RACK1 also contributes directly to adaptive immune-cell development and function. In B cells, RACK1 deficiency impairs B-cell development and function, at least in part through its interaction with and stabilization of the B-cell lineage transcription factor Pax5 [66]. This finding suggests that RACK1 contributes to the maintenance of B-cell identity and fundamental processes underlying humoral immunity. During blood-stage malaria infection, RACK1 also stabilizes STAT3 in CD4^+^ T cells, thereby promoting the differentiation and function of T follicular helper (Tfh) cells. Enhanced Tfh responses support germinal-center formation, antigen-specific IgG production, and protective host immunity [67]. Together, these observations suggest that RACK1 can influence adaptive immune responses through coordinated effects on both B-cell homeostasis and the Tfh–B-cell axis.

Collectively, these findings broaden the immunological functions of RACK1 from the acute organization of signaling complexes to the long-term maintenance, differentiation, and functional competence of immune-cell populations. RACK1 may therefore influence infection outcomes not only by modulating immediate innate signaling responses but also by shaping the cellular composition and adaptive immune capacity of the host.

### Pathogen manipulation of RACK1-dependent signaling

Pathogens can exploit RACK1-dependent signaling complexes to interfere with host antiviral defenses at multiple levels. During bovine ephemeral fever virus (BEFV) infection, RACK1 promotes viral replication by facilitating MAVS degradation through the ubiquitin–proteasome system. Mechanistically, RACK1 increases the expression of the E3 ubiquitin ligase STUB1 and enhances the STUB1–MAVS interaction, thereby promoting MAVS ubiquitination and degradation and suppressing type I interferon signaling [53,68]. During respiratory syncytial virus (RSV) infection, virus-induced activation of AMP-activated protein kinase (AMPK) promotes phosphorylation of RACK1 at Thr50, strengthening its interaction with IRF3 and IRF7 and restricting their phosphorylation, ultimately reducing type I interferon production [48]. Together, these findings indicate that viruses can exploit RACK1-associated complexes to suppress antiviral signaling upstream of interferon production and delay establishment of the antiviral state.

Pathogens can also interfere with RACK1-associated antiviral responses downstream of pathogen sensing, particularly at the levels of interferon receptor signaling and nucleocytoplasmic transport. During measles virus infection, the viral C and V accessory proteins inhibit IFN-α signaling by suppressing IFN-α-induced JAK1 phosphorylation while relatively preserving IFN-γ signaling. This selective inhibition has been linked to viral interference with type I interferon receptor-associated signaling complexes, in which RACK1 normally functions as a scaffold [69,70]. Coronavirus infection provides another example of RACK1-dependent immune evasion. The viral nucleocapsid protein promotes recruitment of phosphorylated PKCα to RACK1, leading to phosphorylation and cytoplasmic redistribution of NUP62 and other FG-repeat nucleoporins [39]. Disruption of nuclear-pore organization impairs nucleocytoplasmic transport and restricts nuclear entry of antiviral transcription factors, thereby broadly suppressing innate immune gene expression.

Pathogens may further exploit RACK1-dependent signaling to regulate host-cell survival and apoptosis, thereby creating conditions favorable for intracellular replication. During infectious bursal disease virus (IBDV) infection, RACK1 interacts with the viral VP5 protein and VDAC2 to form a VP5–VDAC2–RACK1 complex. Formation of this complex attenuates IBDV-induced apoptosis by suppressing the mitochondrial apoptotic pathway and reducing activation of caspase-9 and caspase-3, thereby promoting viral replication [52].

RACK1-associated PKC signaling and cytoskeletal regulation can also be exploited during infection. The influenza A virus M1 protein interacts with RACK1 and can undergo PKC-dependent phosphorylation [71]. Similarly, the Epstein–Barr virus (EBV)-encoded ZEBRA protein interacts with RACK1 and interferes with PKC activation or membrane translocation, thereby affecting host functions including monocyte phagocytosis and inflammatory-mediator production [72]. Evidence from bacterial infection is more limited but suggests a similar principle. During *Shigella flexneri* infection, RACK1 contributes to pathogen-induced actin cytoskeletal remodeling and influences bacterial invasion, intracellular motility, and cell-to-cell spread [73]. These findings suggest that pathogens can co-opt RACK1 as a host scaffold to access signaling, cytoskeletal, and membrane-remodeling processes that support infection.

Collectively, these studies demonstrate that pathogens can exploit or redirect RACK1-associated signaling complexes at multiple stages of infection, including antiviral sensing, interferon signaling, nucleocytoplasmic transport, apoptosis, and cytoskeletal remodeling. However, these mechanisms remain highly pathogen- and context-dependent, and the extent to which they represent broadly conserved infection strategies remains unclear. Representative RACK1-interacting proteins and complexes, together with their reported functions and relevance to infection, are summarized in Table 1.

**Table 1.** Representative RACK1-interacting proteins and complexes in host–pathogen interactions.

Functional category	RACK1-interacting protein / complex	Main function	Infection relevance	Representative pathogen/model	Reference
Signaling / translation	PKCβII	Recruits activated PKCβII to the ribosome and supports signal-dependent translational control	Mechanistic basis for signaling–translation coupling; infection relevance remains indirect	Ribosome/translation model	[74,75]
Signaling / innate immunity	PP2A–IRF3	Promotes IRF3 dephosphorylation and terminates type I IFN signaling	Limits IFN-I signaling after low-titer SeV infection	Low-titer Sendai virus infection / macrophages	[32]
Innate immunity	IFNAR2–STAT1/TYK2/JAK1	Organizes the type I IFN receptor signaling complex and promotes STAT1 activation	Supports IFN-I-mediated antiviral responses	Type I IFN signaling models	[37]
Innate immunity	STING	Promotes STING Golgi aggregation and IRF3-dependent IFN-I activation	Restricts PRV infection	PRV / cGAMP-stimulated PK-15 cells	[76]
Inflammasome	NLRP3 / NEK7	Promotes NLRP3 active conformation and inflammasome assembly	Promotes IL-1β and IL-18 production during infection	*P. multocida*-infected macrophages	[59]
Translation	RACK1–PKCβII–eIF6	Facilitates eIF6 release and 80S ribosome assembly	General translational-control model; not directly tested during infection	Ribosome/translation model	[74]
Translation	ZBP1 / Src	Couples Src signaling to selective β-actin mRNA translation	Mechanistic model for transcript-selective translation; infection relevance remains to be established	Non-infectious neuronal/cell model	[77]
Translation / viral replication	Vigilin / SERBP1	Links viral RNA-associated host factors to RACK1-containing 40S ribosomes	Supports DENV infection and replication	DENV infection model	[78]
Stress signaling	MTK1 / stress-granule components	Regulates stress-responsive p38/JNK signaling through stress-granule sequestration	Potential relevance to infection-associated stress; not directly tested during infection	Cellular stress model: hypoxia, heat shock, or arsenite exposure	[22]
Stress / autophagy	ATG5	Regulates autophagy through the RACK1–ATG5 interaction	Potential stress-adaptation role; not directly tested during infection	Non-infectious autophagy model	[62]
Apoptosis / viral replication	VDAC2 / VP5	Inhibits apoptosis through the VP5–VDAC2–RACK1 complex	Enhances IBDV replication	IBDV-infected avian cells	[52]
Cytoskeletal / adhesion signaling	FAK / PDE4D5	Regulates cell-spreading initiation and cell polarity	Potential relevance to pathogen entry; not directly tested during infection	Cell-spreading/polarity model	[33]

## Ribosome-associated RACK1: bridging signaling and translation

### RACK1 on the 40S ribosome

RACK1 is a prominent component of the surface-exposed head region of the eukaryotic 40S ribosomal subunit. Cryo-electron microscopy and structural studies have shown that RACK1 is positioned near the mRNA exit channel, where it contacts ribosomal proteins uS3, uS9, and eS17, as well as helices 39 and 40 of 18S rRNA. Its ribosome-binding interface is concentrated on one face of the β-propeller, where conserved positively charged residues interact with the negatively charged rRNA backbone and stabilize RACK1 association with the ribosome [11,12,74].

The opposite face and lateral surfaces of the β-propeller remain solvent-exposed and accessible to interacting proteins. Structural analysis by Nielsen et al. showed that many previously identified protein-binding regions of RACK1 remain accessible in the ribosome-bound state, supporting its role as a ribosomal signaling platform [12]. This positioning allows RACK1 to recruit regulatory proteins directly to the translational machinery.

### RACK1’s role in protein translation

RACK1 regulates translation through its association with the 40S ribosomal subunit [27]. Rather than uniformly affecting global translation, ribosome-associated RACK1 can influence translation initiation and the selective translation of specific cellular and viral mRNAs [15,75,79]. RACK1 also interacts with components of the miRNA-induced silencing complex (miRISC) in both nematodes and mammals. Altered RACK1 expression impairs the association of miRNA effector complexes with translating ribosomes, suggesting a role for RACK1 in coupling post-transcriptional repression to the translational machinery [80].

RACK1 can connect kinase signaling to translation initiation through several mechanisms. One involves eukaryotic initiation factor 6 (eIF6), which binds the 60S ribosomal subunit and prevents premature 40S–60S joining. Under pro-translational conditions, ribosome-associated RACK1 recruits activated PKCβII and promotes PKC-dependent phosphorylation of eIF6, including at Ser235 [15,75,79]. Structural studies further support the association of PKC with ribosome-bound RACK1 [45]. Phosphorylation promotes eIF6 release from the 60S subunit, facilitating 80S ribosome assembly and translation initiation [81].

Ribosomal RACK1 also links PKCβII-associated signaling to eIF4E-dependent translation. In hepatocellular carcinoma cells, RACK1 promotes recruitment of PKCβII to the translational machinery and enhances phosphorylation of eIF4E at Ser209. Disruption of RACK1 ribosome binding or inhibition of the RACK1–PKCβII interaction reduces eIF4E phosphorylation [82]. These findings do not establish eIF4E as a direct PKCβII substrate, but instead support a scaffolding role for RACK1 in coupling PKCβII-associated signaling to eIF4E regulation. Ribosome-bound RACK1 is also required for efficient recruitment of eIF4E to the translational machinery and for efficient translation of capped mRNAs [83,84].

RACK1 can further confer transcript selectivity by recruiting signaling proteins to specific messenger ribonucleoprotein complexes. A well-characterized example involves β-actin mRNA and zipcode-binding protein 1 (ZBP1/IGF2BP1). ZBP1 binds β-actin mRNA and maintains it in a translationally repressed state, whereas ribosome-associated RACK1 recruits the β-actin mRNA–ZBP1 complex together with Src kinase to the ribosome [77]. Upon Src activation, phosphorylation of ZBP1 at Tyr396 releases β-actin mRNA from translational repression and permits its local translation. Mutation of RACK1 Tyr246, a Src-interacting and phosphorylation site, disrupts recruitment of ZBP1 and Src to the ribosome and reduces β-actin mRNA translation [85]. These observations illustrate how ribosomal RACK1 can couple upstream signaling to the selective translation of individual transcripts. Whether analogous mechanisms regulate inflammatory or antiviral transcripts during infection remains unclear.

### RACK1 connects stress signaling to translational quality control

RACK1 also participates in ribosome-associated quality control (RQC), which detects aberrant translation events and resolves stalled or collided ribosomes. When elongating ribosomes encounter defective mRNAs or other impediments, prolonged stalling can lead to ribosome collisions. RACK1 forms part of the collided-ribosome interface and cooperates with the E3 ubiquitin ligase ZNF598 to promote ubiquitination of 40S ribosomal proteins, thereby initiating downstream quality-control pathways that terminate aberrant translation and facilitate clearance of defective nascent products [86–88].

RACK1 also links ribosomal stress signaling to co-translational protein quality control through the JNK–eEF1A2 pathway. Under cellular stress, activated JNK is recruited to polysomes through ribosome-associated RACK1 and phosphorylates eEF1A2 at Ser205 and Ser358. This enhances the association of eEF1A2 with newly synthesized polypeptides and promotes their proteasomal degradation [89], providing a direct connection between stress-responsive kinase signaling and nascent-protein quality control.

Ribosomal abnormalities can also be converted into broader cellular stress signals through RACK1-dependent activation of ZAKα. ZAKα monitors translating ribosomes near the mRNA exit channel through interactions with RACK1, ribosomal proteins, rRNA, and mRNA. Reduced ribosome processivity or ribosome collision stabilizes ZAKα on the ribosome, whereas interaction with RACK1 facilitates ZAKα activation and downstream p38/JNK signaling [90,91]. In this way, ribosome-associated RACK1 contributes both to translational quality control and to signaling responses triggered by ribosomal stress.

### RACK1-dependent translation of pathogen proteins

During viral infection, ribosome-associated RACK1 can be exploited to support viral protein synthesis, particularly in translation programs that rely on internal ribosome entry sites (IRESs) or other non-canonical initiation mechanisms. RACK1 has been identified as a host factor required for efficient IRES-dependent translation in several viral systems, and its depletion reduces viral translation [92]. During poliovirus infection, RACK1 deficiency reduces viral plaque size and viral titers and delays host translational shutoff, indicating that efficient poliovirus replication depends on ribosomal RACK1 [93].

RACK1-dependent translational regulation also occurs during DNA virus infection. In poxvirus-infected cells, the viral B1 kinase phosphorylates ribosome-associated RACK1 and promotes translation of post-replicative viral mRNAs containing 5′ poly(A) leaders [94]. This function appears to be independent of RACK1-mediated ribosomal stress signaling [95], indicating a more direct role in translational reprogramming.

Dependence on RACK1, however, varies among viral translation strategies. Although RACK1 enhances translation driven by several viral IRESs, poliovirus IRES-mediated translation shows only moderate dependence on RACK1, whereas translation driven by the cricket paralysis virus (CrPV) intergenic-region IRES is largely RACK1-independent [92,93]. Even within CrPV, the 5′ IRES requires RACK1 whereas the intergenic-region IRES does not, indicating that RACK1 dependence is determined by the mechanism of translation initiation rather than simply by viral identity.

### RACK1 and host translational reprogramming

Infection is frequently accompanied by global translational repression, while selected host transcripts remain efficiently translated to support stress adaptation and immune responses [96–99]. Whether ribosome-associated RACK1 specifically directs the translation of host immune-response transcripts during infection remains unclear.

Evidence from noninfectious models shows that ribosomal RACK1 can selectively regulate host mRNA translation. Depletion of ribosome-associated RACK1 alters the translation of specific transcripts, including LC3 and Bcl-xL, whose increased association with polysomes contributes to non-canonical autophagy [26]. This demonstrates that changes in ribosomal RACK1 can reshape translational output without uniformly affecting global protein synthesis.

Studies of vaccinia virus (VacV)-induced host shutoff further illustrate the limits of RACK1 dependence. Several host transcripts retain or increase their polysome association during infection, with JUN representing a prominent example. Although viral mRNA translation requires RACK1 [95], JUN production remains largely RACK1-independent, and broader host transcriptional and translational reprogramming is largely preserved in RACK1-deficient cells [5]. Thus, current evidence does not yet establish RACK1 as a general regulator of infection-induced host translational reprogramming, and the identity of any immune-related transcripts selectively controlled by ribosomal RACK1 remains to be defined.

## What determines RACK1 localization?

RACK1 association with the ribosome is stable but dynamic. Biochemical studies of human RACK1 have shown that free RACK1 can reassociate with mature 40S subunits, whereas ribosome-bound RACK1 remains stably associated for hours *in vitro* [11,83]. Conversely, a ribosome-binding-defective RACK1 mutant accumulates preferentially in the soluble fraction and has a shorter half-life than ribosome-associated RACK1 [83]. Ribosome association therefore influences both the cellular distribution and stability of RACK1, whereas the free RACK1 pool appears to be more transient.

Competition among binding partners and post-translational modifications may further shape RACK1 complex formation and functional output [7,48,94]. Different proteins can compete for RACK1 binding, while modifications such as phosphorylation alter its interactions and signaling functions [29,31,37]. Current evidence, however, more strongly supports regulation of partner selection and complex assembly than direct switching between ribosome-bound and ribosome-free states. How infection or cellular stress alters RACK1 ribosome occupancy and redistribution among competing complexes remains unclear.

## A proposed signaling–ribosome model for context-dependent RACK1 functions during infection

Based on the evidence discussed above, RACK1 functions during infection can be considered in three interconnected modes. In Mode 1, extraribosomal signaling, RACK1 organizes host signaling complexes involved in inflammatory and antiviral responses. In Mode 2, ribosome-dependent pathogen exploitation, pathogens use RACK1-containing ribosomes or ribosome-associated complexes to support viral gene expression and replication. Mode 3, the signaling–translation interface, represents a less well-established state in which infection- or stress-induced signaling converges on ribosome-associated RACK1 to alter translational output. These modes may coexist or shift during infection according to RACK1 localization, interacting partners, post-translational modifications, cell type, pathogen, and infection stage (Figure 3).

**Figure 3.**
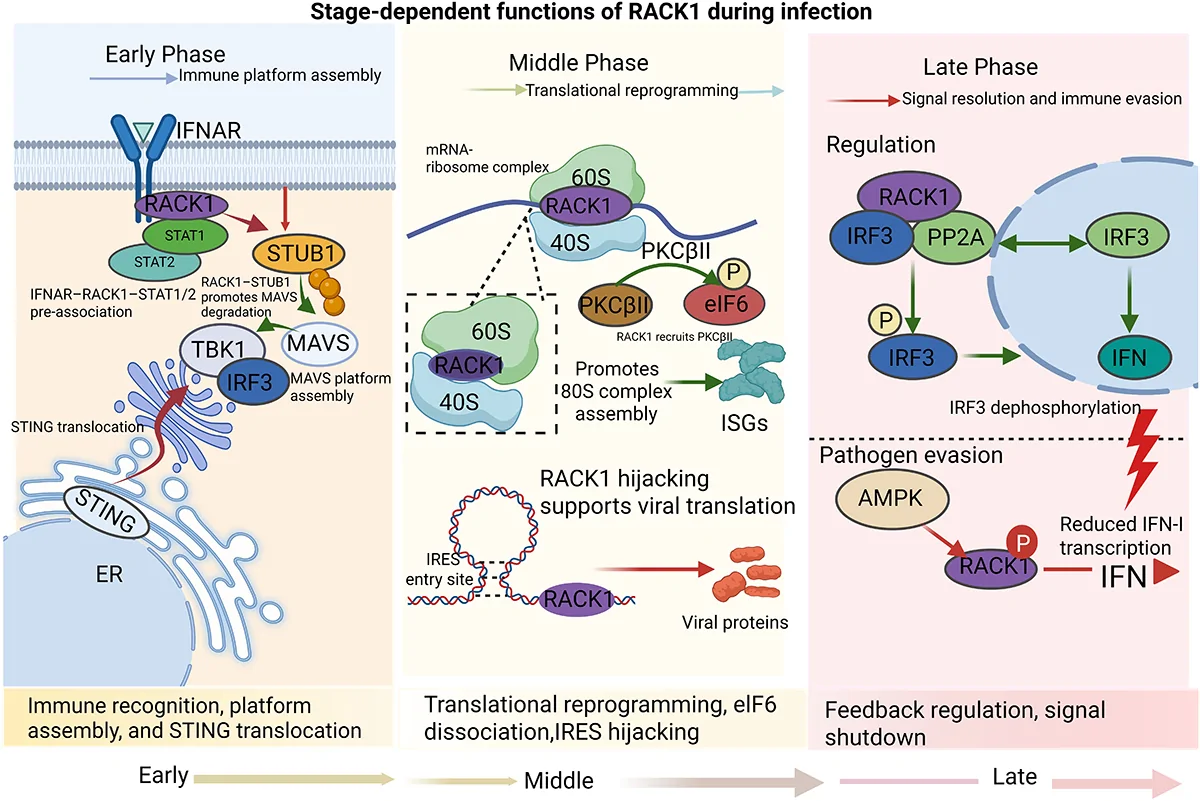
Proposed stage-associated functions of RACK1 during viral infection. RACK1 may exert distinct functions at different stages of viral infection. During the early phase, RACK1 participates in antiviral signaling complexes, including IFNAR-associated STAT recruitment, MAVS-dependent signaling, and STING trafficking. During the intermediate phase, ribosome-associated RACK1 supports translational regulation through PKCβII-dependent eIF6 phosphorylation, eIF6 release, 80S ribosome assembly, and RACK1-dependent viral translation. During the late phase, RACK1-containing complexes may contribute to signal termination through PP2A-mediated IRF3 dephosphorylation, whereas AMPK-dependent phosphorylation of RACK1 can restrain IRF3/IRF7 activation and type I interferon production. The model illustrates how changes in RACK1 localization and interacting partners may shift its functional contribution during infection.

An example of the extraribosomal signaling mode is the RACK1–PP2A–IRF3 complex. Following Sendai virus infection, RACK1 facilitates the association of protein phosphatase 2A (PP2A) with activated IRF3, promoting IRF3 dephosphorylation and termination of type I interferon signaling [32]. This mechanism is particularly evident after low-titer infection, when activated IRF3 returns to a resting state through dephosphorylation rather than degradation. Consistently, PP2A-deficient macrophages display enhanced type I interferon responses following Sendai virus challenge. This example illustrates how RACK1 can control the magnitude and duration of antiviral signaling independently of the translational machinery.

A distinct ribosome-dependent mechanism operates during dengue virus (DENV) infection. RACK1 is incorporated into the DENV replication complex and supports infection through its association with the 40S ribosomal subunit. Proteomic and functional studies identified the RNA-binding proteins Vigilin and SERBP1 as RACK1-associated host-dependency factors that also interact with the DENV genome. Disrupting the interaction of either protein with RACK1 markedly impairs DENV infection even when viral RNA binding is retained [78]. Ribosome-associated RACK1 may therefore organize viral RNA-binding host factors into a functional complex that supports DENV replication.

Mode 3 represents a more integrated relationship between signaling and translational control. Studies of vaccinia virus (VacV) show that RACK1-containing ribosomes are dynamically remodeled during infection. Viral mRNA translation depends on RACK1 and eIF3, whereas selected host transcripts such as JUN remain largely RACK1-independent, as discussed in RACK1 and host translational reprogramming. Cryo-electron microscopy further revealed that eIF3-bound 40S ribosomes from infected cells exhibit an expanded rotational range of the RACK1-containing 40S head, indicating the formation of structurally distinct ribosomal states associated with selective translation initiation [5]. Together with the signaling-to-translation mechanisms described in RACK1 and host translational reprogramming and RACK1 connects stress signaling to translational quality control and , these observations provide a plausible framework in which infection-induced signaling could converge on ribosome-associated RACK1.

Direct evidence for such a complete signaling–RACK1–translation pathway during infection remains limited. In vaccinia virus infection, RACK1 knockout reduces late-stage JNK activation, yet RACK1 is not required for JNK recruitment to ribosomes, and pharmacological JNK inhibition does not impair viral protein synthesis [94]. Thus, the translational role of RACK1 in poxvirus infection can be experimentally separated from its role in ribosome-associated stress signaling. Mode 3 should therefore be regarded as a mechanistically plausible but incompletely validated model rather than an established general mechanism. Representative pathogen-specific RACK1 complexes and their functional consequences are summarized in Table 2.

**Table 2.** Representative roles of RACK1 and RACK1-associated complexes in viral and bacterial infections.

Pathogen/model	RACK1-related partner or complex	Infection stage/process	Functional outcome	Reference
LCDV	RACK1–VDAC2 receptor-related complex	Viral entry	Facilitates viral entry and increases cellular susceptibility	[100]
Poliovirus	Ribosome-associated RACK1	IRES-mediated translation	Enhances viral IRES-dependent translation and supports viral production	[93]
HCV	RACK1–NS5A / ATG14L-related complex	Replication compartment formation	Supports double-membrane vesicle formation and viral RNA replication	[101]
Poxvirus	Phosphorylated ribosome-associated RACK1	Late viral mRNA translation	Promotes translation of post-replicative viral transcripts with 5′ poly(A) leaders	[94]
BEFV	RACK1–STUB1–MAVS axis	Antiviral signaling suppression	Promotes MAVS degradation and suppresses type I IFN signaling	[68]
RSV	Phosphorylated RACK1–IRF3/IRF7 complex	IFN induction phase	Limits IRF3/IRF7 phosphorylation and reduces IFN-I production	[102]
Measles virus	IFNAR1–RACK1–STAT1 complex	IFN receptor signaling	Selectively attenuates IFN-α signaling	[69]
Coronavirus	RACK1–PKCα–NUP62-related axis	Nuclear transport of antiviral transcription factors	Restricts nuclear entry of antiviral transcription factors and suppresses innate immune gene expression	[39]
IBDV	VP5–VDAC2–RACK1 complex	Apoptosis regulation	Inhibits apoptosis and prolongs the intracellular replication window	[52]
Influenza A virus	M1–RACK1 interaction	Virion assembly/release	Supports viral particle release and membrane-associated assembly events	[71]
PRRSV	RACK1–ERK1/2/ ITGB3–RACK1–NF-κB axis	Viral replication and inflammatory feedback	Promotes PRRSV infection and NF-κB-related positive feedback	[46]
PIgR-mediated bacterial internalization model	Src/FAK/ERK signaling module associated with RACK1-related scaffolding	Bacterial internalization	Supports actin remodeling required for bacterial uptake	[103]
*Shigella flexneri*	RACK1-associated actin remodeling machinery	Invasion, intracellular motility, cell-to-cell spread	Promotes bacterial invasion and dissemination	[73]
*Mycobacterium tuberculosis*	EST12–RACK1–JNK–AP-1–Myc axis	Antibacterial inflammatory response	Enhances IL-6, TNF-α, iNOS expression and NO production	[104]

The relative contribution of these modes is likely determined by the partitioning of RACK1 between ribosome-bound and extraribosomal pools, together with the availability of interaction partners and regulatory modifications. Such dynamic partitioning may help explain why RACK1 supports antiviral or inflammatory responses in some settings while facilitating pathogen translation, replication, or immune evasion in others. RACK1 function during infection may therefore be better understood as the context-dependent output of a dynamic signaling–ribosome axis rather than as uniformly host-protective or pathogen-supportive.

Testing this model will require direct analysis of RACK1 functional partitioning during infection. Measurements of RACK1 ribosome occupancy, combined with ribosome profiling after RACK1 depletion, could identify RACK1-dependent translational outputs. Comparative interactome analysis of ribosome-bound and ribosome-free RACK1, together with phosphoproteomic profiling, could reveal how infection alters partner selection and post-translational regulation. Extending these approaches to primary and immune-cell populations will be important for determining whether RACK1 selectively controls inflammatory, antiviral, stress-response, or pathogen-restriction transcripts.

## Therapeutic implications of RACK1 in infection

Because RACK1 participates in both host signaling and ribosome-associated translation, global inhibition of RACK1 may disrupt essential cellular functions. Therapeutic strategies may therefore be more feasible when directed toward pathogen-favorable RACK1 interactions or specific RACK1-containing complexes. Several experimental compounds provide proof-of-concept for this approach. SD-29 and its analog SD-29–14 target functional regions of RACK1 and inhibit HSV-1 gene expression and replication [105]. SB2960 exhibits broad-spectrum antiviral activity by binding RACK1 and altering stress-granule composition [106]. Bigelovin, a sesquiterpene lactone, targets Cys168 of RACK1 and disrupts the RACK1–NLRP3 interaction, thereby suppressing NLRP3 inflammasome activation and associated tissue injury [107]. Although these studies support the pharmacological tractability of RACK1-associated interfaces, efficacy, selectivity, and safety remain to be established in clinically relevant models.

The expression, subcellular distribution, post-translational modification, and interaction profile of RACK1 can change in response to infection and immune activation [10,37,48,108]. These dynamic features raise the possibility that specific RACK1 states could serve as biomarkers of infection-associated inflammation, immune evasion, or therapeutic response. However, their disease specificity, reproducibility, and clinical utility have not yet been established.

Pathogen-derived RACK1 has also been explored as a vaccine antigen. Recombinant *Toxoplasma gondii* RACK1 (rTgRACK1) induces antigen-specific immune responses in mice [109,110]. Intranasal immunization with rTgRACK1 increases mucosal secretory IgA, IFN-γ, IL-2, and an IgG2a-dominant antibody response and provides partial protection against challenge with the virulent RH strain. These findings remain limited to experimental mouse models and should be regarded as proof-of-concept rather than evidence of an established vaccine strategy. Given the high evolutionary conservation of RACK1 [10,111], potential cross-reactivity with host RACK1 and the feasibility of pathogen-specific targeting require careful evaluation. Representative therapeutic, biomarker, and vaccine-related applications of RACK1 are summarized in Table 3.

**Table 3.** Therapeutic, biomarker, and vaccine-related applications of RACK1 in infection.

Strategy	Example	RACK1-related target/mechanism	Potential significance	Reference
Host-directed antiviral intervention	SD-29 / SD-29–14	Targeting RACK1 functional interfaces	Inhibits HSV-1 gene expression and viral replication; enhances acyclovir efficacy	[105]
Stress granule-related antiviral strategy	SB2960	Binding RACK1 and remodeling stress granule composition	Shows broad-spectrum antiviral potential by modulating host stress responses	[106]
Anti-inflammatory intervention	Bigelovin	Targeting RACK1 Cys168 and disrupting RACK1–NLRP3 interaction	Suppresses NLRP3 inflammasome activation and inflammatory cytokine release	[107]
Biomarker exploration	RACK1 expression, localization, PTMs, interaction network	Monitoring RACK1 molecular state during infection	Reflect infection stage, inflammatory intensity, immune evasion status, or treatment response	[46]
Vaccine antigen exploration	rTgRACK1 from *Toxoplasma gondii*	Pathogen-derived RACK1-related antigen	Preclinical proof-of-concept; induces antigen-specific immune responses, partial protection in mice, and Th1-type immune responses	[112]

## Discussion

Since its identification as a receptor for activated PKCβII, RACK1 has emerged as a multifunctional scaffold with diverse binding partners and cellular functions [6,53]. This versatility is particularly relevant to host–pathogen interactions, where RACK1 participates in inflammatory and antiviral signaling, inflammasome activation, ribosome-associated translation, and pathogen-driven translational reprogramming [7,10,41,92,93].

A major determinant may be the partitioning of RACK1 among distinct cellular compartments and protein complexes. RACK1 is found in ribosome-associated, cytosolic, membrane-proximal, stress-granule-associated, and other functional pools [7,10,11,113]. Ribosome-associated RACK1 is closely linked to selective translation, non-canonical translation initiation, and viral protein synthesis, whereas extraribosomal RACK1 more commonly participates in receptor-proximal signaling, inflammatory responses, cytoskeletal regulation, and interferon signaling [39,102]. These functional states are not fixed, and redistribution of RACK1 may alter the signaling or translational outputs available to both host and pathogen.

Partner availability provides an additional layer of regulation. Although RACK1 is broadly expressed, its interaction network varies with cell type, pathogen, infection stage, and signaling environment [10,101]. In some settings, RACK1 associates with host defense components such as STING, IFNAR, STAT1, or NLRP3; in others, pathogens redirect RACK1 toward complexes that support viral translation, replication, immune evasion, apoptosis suppression, or bacterial invasion [37,53,60,76,101]. Competition among binding partners and post-translational modifications may further reshape these complexes [7,29,31,37,48,94]. Thus, the biological role of RACK1 is likely determined less by its abundance alone than by its localization, modification state, and interaction profile.

This framework also helps explain apparently conflicting observations across infection models. Rather than functioning as a simple pro- or anti-infective factor, RACK1 may act as a dynamic signaling–ribosome hub whose output changes according to the complexes it enters. Pathogen proteins, immune signaling molecules, ribosomal factors, and stress-response components may compete for overlapping RACK1 interaction surfaces, redirecting the scaffold toward distinct functional programs [39,52,102]. Defining these infection-specific RACK1 states will therefore be important for distinguishing mechanisms that support host defense from those exploited by pathogens.

From a therapeutic perspective, RACK1 is unlikely to be suitable for indiscriminate inhibition or activation because of its roles in translation, signaling, cytoskeletal regulation, and immune-cell homeostasis. More selective approaches could target pathogen-favorable RACK1 interfaces, disease-associated post-translational modifications, or specific RACK1-containing complexes while preserving essential cellular functions. Such strategies are consistent with emerging evidence that discrete RACK1 interactions can be pharmacologically modulated.

RACK1 may therefore be best viewed as a spatially organized regulatory hub linking signaling, translation, and pathogen-driven cellular reprogramming. This perspective provides a mechanistic explanation for its context-dependent roles in infection and suggests that future studies should focus on how RACK1 localization, ribosome association, post-translational modification, and partner selection change over the course of infection. Resolving these dynamic states will be essential for determining when RACK1 contributes to host defense and when it becomes a dependency exploited by pathogens.
